# Safety, Tolerability and Pharmacokinetics of the Serotonin 5-HT6 Receptor Antagonist, HEC30654, in Healthy Chinese Subjects

**DOI:** 10.3389/fphar.2021.726536

**Published:** 2021-08-19

**Authors:** Xiaojiao Li, Lei Gao, Jingrui Liu, Hong Zhang, Hong Chen, Lizi Yang, Min Wu, Cuiyun Li, Xiaoxue Zhu, Yanhua Ding, Li Sun

**Affiliations:** ^1^ Phase I Clinical Trial Unit, The First Hospital of Jilin University, Changchun, China; ^2^ Nanguan District Maternal and Child Health and Family Planning Service Center of Changchun, Changchun, China; ^3^ Department of Neurology and Neuroscience Center, The First Hospital of Jilin University, Changchun, China

**Keywords:** HEC30654, pharmacokinetics, safety, phase I, 5-HT6 receptor antagonist

## Abstract

**Background and Objective:** HEC30654 is a selective 5-HT6 receptor antagonist that was safe and well-tolerated in preclinical models of Alzheimer’s disease. The objective of this double-blind, randomized, placebo-controlled clinical trial was to evaluate the safety, tolerability, and pharmacokinetic profile of HEC30654 after single ascending doses in healthy Chinese subjects.

**Methods:** Healthy volunteers received a single oral dose of HEC30654 (5, 10, 15, 30, 60 mg). Safety and tolerability assessments included adverse events, vital signs, and findings on electrocardiograms, electroencephalograms, physical examination, and clinical laboratory tests. Pharmacokinetic analysis of HEC30654 and its major metabolite HEC93263 were conducted in blood, urine, and fecal samples.

**Results:** Single doses of HEC30654 up to 30 mg were generally safe and well tolerated, but dose escalation was terminated early as the 60 mg HEC30654 treatment group met the pre-defined stopping rules specified in the protocol. Median t_max_ of HEC30654 was 6 h (range, 4–12 h), t_1/2_ of 10–60 mg HEC30654 ranged from 52.1 to 63.8 h. Exposure to HEC30654 across the dose range explored in this study increased more than in proportion to dose. Metabolism of HEC30654 to HEC93263 was slow (<10%), and HEC30654 was mainly eliminated unchanged through feces.

**Conclusion:** Single doses of HEC30654 up to 30 mg were generally safe and well tolerated. Based on preclinical efficacy in various models of cognition, HEC30654 may represent a therapeutic option for symptomatic treatment of cognitive disorders.

## Introduction

Alzheimer’s disease (AD) is a chronic neurodegenerative disease that manifests as a progressive impairment of memory and cognitive function, behavioral changes, and psychiatric symptoms, including depression and psychosis. The pathogenesis of AD remains to be elucidated, but involves amyloid plaques, neurofibrillary tangles, and profound neuronal loss (Upton et al., 2008; Ramírez, 2013; Folch et al., 2016; Sun et al., 2018). Currently available treatments for AD, such as acetylcholinesterase inhibitors (donepezil, rivastigmine, and galantamine) and the N-methyl-d-aspartate (NMDA) receptor antagonist memantine, help manage patients’ symptoms but do not change or delay progression of the disease (Wicke et al., 2015; Ferrero et al., 2017). There remains a critical unmet clinical need for disease-modifying therapies (DMTs) that halt or slow the progression of AD. In preclinical studies, 5-HT6 receptor reportedly modulated the activity of cholinergic systems, and there is evidence suggesting that the blockade of 5-HT6 receptors induces acetylcholine release, it became apparent that 5-HT6 antagonism may be a promising approach to restore acetylcholine levels in a deteriorated cholinergic system (Ramírez, 2013).

Recently, 5-HT_6_ receptor antagonists have emerged as potential treatment strategies in AD, with some 5-HT_6_ receptor antagonists, such as Lu-AE58054 (idalopirdine) and masupirdine (SUVN-502), being investigated in Phase II or Phase III clinical trials (Wilkinson et al., 2014; Khoury et al., 2018; Frölich et al., 2019; Nirogi et al., 2019; Nirogi et al., 2020). In these clinical studies, 5-HT_6_ receptor antagonists were usually used as an adjunct to combination therapy (cholinesterase inhibitor and memantine) in patients with AD (Khoury et al., 2018). The results shown that idalopirdine was not effective for AD patients as a whole, although idalopirdine might be more effective at high doses and in moderate AD subgroups (Matsunaga et al., 2019). In a triple therapy with masupirdine added to background dual combination treatment with donepezil and memantine, the treatment significantly reduced the behavioral and psychological symptoms, with positive effect on cognition (Nirogi et al., 2020).

HEC30654 is a 5-HT_6_ receptor antagonist with higher receptor affinity (IC50 = 1.1 nM) and antagonistic activity (IC50 = 15 nM) than LuAE58054 (receptor affinity IC50 = 2.2 nM; antagonistic activity IC50 = 26 nM). The metabolite of HEC30654, HEC93263, is a weak 5-HT_6_ receptor antagonist, with an IC50 of 1.7 μM HEC30654 has weak affinity for histamine (H_1_, H_2_) receptors, cholinergic (M_1_, M_2_, M_3_) receptors, and 5-HT (5-HT_1A_, 5-HT_1B_, 5-HT_7_) receptors, and a strong affinity for 5-HT_2A_ and 5-HT_2B_ receptors; however, HEC30654 has weak antagonistic activity against 5-HT_2A_ and 5-HT_2B_ receptors, with an IC50 of 0.12 and 0.99 μM, respectively. In pre-clinical evaluations, rodent behavioral testing showed HEC30654 significantly improved scopolamine-induced cognitive dysfunction with greater efficacy than LuAE58054.

In rats and dogs (beagles), HEC30654 had a terminal half-life (t_1/2_) of 4–8 h after intravenous administration, and bioavailability of HEC30654 after oral administration was 12.2% or 51%, respectively. In animals and humans, HEC30654 had a plasma protein binding rate of >93%. Previous studies revealed that phase 1 metabolism of HEC30654 may involve CYP3A4, CYP2D6, and CYP2B6. In rats, mean total recovery of radioactivity in urine and fecal samples after a single oral administration of 89.7 μCi/6.04 mg/kg [^14^C] HEC30654 was 95.0%, with urine and feces accounting for 2.46 and 91.0%, respectively.

The present study aims to evaluate the safety, tolerability, and PK profile of HEC30654 after single ascending doses in healthy Chinese subjects.

## Materials and Methods

### Study Subjects

Healthy Chinese volunteers were eligible to participate in this study. Key inclusion criteria were: 1) males and females (not pregnant or lactating) aged 18–45 years; 2) body mass index (BMI) 18–28 kg/m^2^ (body weight: males, ≥ 50 kg, females, 45 kg); and 3) physical examination, medical history, and clinical laboratory test results showed no clinically significant abnormal findings at screening. Key exclusion criteria were: 1) long-term history of smoking or alcohol and/or drug abuse; 2) use of any medication during the 14 days prior to the initial dose of study drug or while participating in the study; 3) blood donation during the 3 months prior to the study; 4) exposure to any CYP3A4, P-gp or Bcrp inducers or inhibitors during the 3 months prior to the initial dose of study drug; or 5) participation in a clinical trial of another investigational drug during the 3 months prior to this study.

### Study Design

This single-center clinical trial was conducted at the Jilin University First Affiliated Hospital-Phase I Clinical Research Center, Changchun City, China between Nov 21, 2018 and Jul 17, 2019. The clinical study protocol was reviewed and approved by the Ethics Committee at the Jilin University First Affiliated Hospital-Clinical Research Institute. The clinical trial was registered at http://www.chinadrugtrials.org.cn/(registration No. CTR20181499, registration date: 22/Nov/2018) and was conducted in accordance with the World Medical Congress Declaration of Helsinki and Good Clinical Practice guidelines. All study subjects provided written informed consent.

This single ascending dose, double-blind, randomized, placebo-controlled clinical trial investigated the safety, tolerability, and PK of HEC30654 in healthy subjects. The study planned to randomize eligible healthy subjects to one of seven treatment groups. In the first treatment group, three sentinel subjects were to receive a single oral dose of 5 mg HEC30654. In the six remaining treatment groups, ten subjects were to receive a single oral dose of 10, 15, 30, 60, 90 or 120 mg HEC30654 (n = 8 per group) or matching placebo (n = 2 per group) after an overnight fast.

Dose escalation was dependant on the safety and tolerability of the previous doses defined according to the National Cancer Institute Common Terminology Criteria for the Classification of Adverse Events (NCI CTCAE) v.4.03. Dose escalation was stopped based on the occurrence of the following drug-related adverse events (AEs): >50% of subjects in the previous treatment group experienced Grade ≥2 drug-related toxicities, or >25% of subjects in the previous treatment group experienced Grade 3–4 drug-related toxicities, or one drug-related serious adverse event (SAE).

The trial was terminated early with subjects in five treatment groups receiving a single dose of 5, 10, 15, 30, or 60 mg HEC30654.

### Safety Assay

Safety and tolerability, including AEs, vital signs (body temperature, blood pressure at rest, heart rate, and respiratory rate), and findings on electrocardiograms, electroencephalograms, physical examination, and clinical laboratory tests (biochemistry, hematology, urinalysis, coagulation, and immune globulin) were evaluated according to NCI-CTCAE, 4.03. Incidence, severity, and relationship of AEs to study drug were recorded. Tolerability was evaluated on Day 2 and Day 6 for subjects administered 5, 10, 15, or 30 mg HEC30654, and Day 2, Day 6, and Day 10 for subjects administered 60 mg HEC30654.

### PK Analysis

Blood samples (4 ml each) were collected *via* an indwelling intravenous angiocatheter into K_2_EDTA containing tubes. For subjects administered 5, 10, 15, or 30 mg HEC30654, blood samples were collected at 0 h (pre-dose) and 0.5, 1, 1.5, 2, 4, 6, 8, 10, 12, 24, 48, 72, 96, and 120 h after dosing. For subjects administered 60 mg HEC30654, according to the t_1/2_ and T_max_ of previous dosages, the time points were adjusted to 0 h (pre-dose) and 1, 2, 4, 6, 8, 10, 12, 24, 48, 72, 96, 120, 144, 168, 192, and 216 h after dosing. After discarding the first 0.5–1 ml of blood, samples were centrifuged at 1,800 *g* for 10 min at 4°C, and plasma was stored as two equal aliquots in polypropylene tubes at −80°C until analysis. For subjects administered 60 mg HEC30654, urine and fecal samples were collected during the following time intervals: 0, 0–4, 4–8, 8–12, 12–24, 24–48, 48–72, 72–96, 96–120, 120–144, 144–168, 168–192, 192–216 h (urine) and 0–216 h (feces).

The concentrations of HEC30654 and its metabolite HEC93263 in plasma and feces were determined using an ExionLC HPLC system (AB Sciex, Toronto, Ontario, Canada) equipped with an AB 4000 QTrap MS detector (AB Sciex, Toronto, Ontario, Canada). The concentrations of HEC30654 and its metabolite HEC93263 in urine were determined with a Nexera X2 HPLC system (Shimadzu, Tokyo, Japan) equipped with an API 4000 MS detector (AB Sciex, Toronto, Ontario, Canada). The calibration ranges of the assays for HEC30654 and HEC93263 were 0.500–500 ng/ml in plasma, 2.00–2,000 ng/ml in urine, and 5.00–5,000 ng/ml (HEC30654) and 1.00–1,000 ng/ml (HEC93263) in feces. For HEC30654 and HEC93263 in plasma, urine and feces, accuracies were 3.33–12.8%, –11.93–6.67% and –6.00–8.88%, and precision was within 6.02, 10.5 and 9.10% CV, respectively.

### Statistical Analysis

Standard non-compartmental methods using WinNonlin version 6.4 (Certara United States of America Inc,.) were used to analyze plasma PK data, including peak plasma concentration (C_max_), time to peak plasma concentration (T_max_), area under the plasma concentration–time curve from time 0 to the last timepoint with a quantifiable concentration (AUC_0-t_), AUC from time 0 to infinity (AUC_0-∞_), t_1/2_, clearance (CL/F), and apparent volume of distribution (Vz/F).

Statistical analysis was performed using SAS software, version 9.4 (SAS Institute Inc., United States). Continuous variables were reported as number, mean with standard deviation, median, maximum, and minimum. Categorical variables, were reported as frequency and percentage. Dose proportionality was explored using a regression power model, relating log-transformed C_max_ and AUC and log-transformed dose.

## Results

### Baseline Characteristics

This study included a total of 43 healthy subjects enrolled into five treatment groups. Baseline demographic characteristics were well balanced across the treatment groups (Table 1). All subjects completed the study and were included in the safety, tolerability, and PK analyses.

**TABLE 1:** Baseline demographic characteristics of study subjects.

	5 mg HEC30654 (n = 3)	10 mg HEC30654 (n = 8)	15 mg HEC30654 (n = 8)	30 mg HEC30654 (n = 8)	60 mg HEC30654 (n = 8)	Placebo (n = 8)
Age, y	36.0 ± 8.19	33.8 ± 5.06	35.1 ± 8.44	32.8 ± 6.67	37.6 ± 2.20	30.8 ± 5.99
Gender, n (%)						
Male	2 (66.7)	4 (50.0)	4 (50.0)	4 (50.0)	4 (50.0)	5 (62.5)
Female	1 (33.3)	4 (50.0)	4 (50.0)	4 (50.0)	4 (50.0)	3 (37.5)
Height, cm	165.4 ± 4.84	164.4 ± 3.90	164.1 ± 7.79	165.9 ± 10.2	163.6 ± 4.67	167.4 ± 11.2
Weight, kg	70.1 ± 10.1	65.0 ± 8.16	60.2 ± 6.89	65.2 ± 12.2	61.0 ± 8.66	65.3 ± 11.1
BMI, kg/m^2^	25.7 ± 2.08	24.0 ± 3.16	22.4 ± 1.77	23.3 ± 2.12	22.8 ± 2.60	23.0 ± 2.78

### Safety and Tolerability

Treatment-emergent AEs (TEAEs) were reported in 33.3, 50.0, 25.0, 12.5, 100, and 37.5% of subjects, and drug-related TEAEs were reported in 33.3, 37.5, 12.5, 12.5, 100, and 25% of subjects administered 5, 10, 15, 30, or 60 mg HEC30654 or placebo, respectively. No dose-dependent TEAEs were observed (Table 2).

**TABLE 2:** Treatment-related adverse events.

AEs	5 mg	10 mg		15 mg		30 mg		60 mg		Placebo
	HEC30654	HEC30654	Placebo	HEC30654	Placebo	HEC30654	Placebo	HEC30654	Placebo	
	(N = 3)	(N = 8)	(N = 2)	(N = 8)	(N = 2)	(N = 8)	(N = 2)	(N = 8)	(N = 2)	(N = 8)
**All AEs**	**1 (33.3%)**	**4 (50.0%)**	**0**	**2 (25.0%)**	**1 (50.0%)**	**1 (12.5%)**	**0**	**8 (100%)**	**2 (100%)**	**3 (37.5%)**
**Drug related AEs**	**1 (33.3%)**	**3 (37.5%)**	**0**	**1 (12.5%)**	**0**	**1 (12.5%)**	**0**	**8 (100%)**	**2 (100%)**	**2 (25%)**
Lymphocytopenia	0	0	0	0	1 (50.0%)	0	0	8 (100%)	2 (100%)	3 (37.5%)
Leukopenia	0	0	0	0	1 (50.0%)	0	0	7 (87.5%)	1 (50.0%)	2 (25.0%)
Elevated C-reactive protein	0	0	0	0	0	0	0	6 (75.0%)	2 (100%)	2 (25.0%)
Neutropenia	0	0	0	0	0	0	0	5 (62.5%)	1 (50.0%)	1 (12.5%)
Elevated alanine aminotransferase	0	1 (12.5%)	0	0	0	1 (12.5%)	0	0	1 (50.0%)	1 (12.5%)
Elevated procalcitonin	0	0	0	0	0	0	0	2 (25.0%)	0	0
Elevated aspartate aminotransferase	0	1 (12.5%)	0	0	0	0	0	0	1 (50.0%)	1 (12.5%)
Neutrophilic granulocytosis	1 (33.3%)	0	0	0	1 (50.0%)	0	0	0	0	1 (12.5%)
Leukocytosis	1 (33.3%)	0	0	0	0	0	0	0	0	0
Thrombocytopenia	0	1 (12.5%)	0	0	0	0	0	0	0	0
Fever	0	0	0	0	0	0	0	8 (100%)	2 (100%)	2 (25.0%)
Fatigue	0	0	0	0	0	0	0	1 (12.5%)	0	0
Headache	0	0	0	0	0	0	0	6 (75.0%)	2 (100%)	2 (25.0%)
Myalgia	0	0	0	0	0	0	0	6 (75.0%)	1 (50.0%)	1 (12.5%)
Arthralgia	0	0	0	0	0	0	0	0	1 (50.0%)	1 (12.5%)
Nausea	0	0	0	0	0	0	0	2 (25.0%)	0	0
Diarrhea	0	0	0	0	1 (50.0%)	0	0	0	0	1 (12.5%)
Dyspepsia	0	0	0	1 (12.5%)	0	0	0	0	0	0
Hypoalbuminemia	0	0	0	0	0	0	0	1 (12.5%)	0	0
Hypokalemia	0	0	0	0	1 (50.0%)	0	0	0	0	1 (12.5%)
Hypochloremia	0	0	0	0	1 (50.0%)	0	0	0	0	1 (12.5%)
Hyponatremia	0	0	0	0	1 (50.0%)	0	0	0	0	1 (12.5%)
Urinary tract infection	0	0	0	0	0	0	0	0	1 (50.0%)	1 (12.5%)
Upper respiratory infection	0	1 (12.5%)	0	0	0	0	0	0	0	0
Rash	0	1 (12.5%)	0	0	0	0	0	0	0	0
Pruritus	0	1 (12.5%)	0	0	0	0	0	0	0	0
Hand bone fracture	0	0	0	1 (12.5%)	0	0	0	0	0	0
Limb crush injury	0	0	0	1 (12.5%)	0	0	0	0	0	0
Nail damage	0	0	0	1 (12.5%)	0	0	0	0	0	0

AE, adverse event; data are n (%). The bold values were AEs or drug related AEs.

Single doses of HEC30654 up to 30 mg were generally safe and well tolerated. 100% of subjects administered 60 mg HEC30654 reported TEAEs, all of which were assessed as related to study drug. TEAEs most frequently reported by subjects administered 60 mg HEC30654 included lymphocytopenia (100%), leukopenia (87.5%), elevated C-reactive protein (75.0%), neutropenia (62.5%), fever (100%), headache (75.0%), and myalgia (75.0%). 100% of subjects in the matching placebo group reported TEAEs (100%), all of which were assessed as related to study drug. TEAEs most frequently reported by subjects administered matching placebo included lymphocytopenia (100%), leukopenia (50.0%), elevated C-reactive protein (100%), neutropenia (50%), fever (100%), headache (100%), and myalgia (50%). Most of the TEAEs reported by subjects administered 60 mg HEC30654 or matching placebo were not reported by subjects in the other treatment groups. There was no clinically significant changes for vital signs, electrocardiograms and electroencephalograms throughout the study for each group.

All the subjects in 60 mg group occurred grade ≥2 drug related AEs, and three of them occurred grade ≥3 drug related AEs (Table 3). As the 60 mg HEC30654 treatment group met the pre-defined stopping rules specified in the protocol, the study was terminated early.

**TABLE 3:** Grade ≥ 2 treatment-related adverse events in subjects administered 60 mg HEC30654.

AEs	60 mg HEC30654 (N = 8)		Matching placebo (N = 2)	
	CTCAE≥2	CTCAE≥3	CTCAE≥2	CTCAE≥3
All AEs	8 (100%)	3 (37.5%)	2 (100%)	0
Lymphocytopenia	8 (100%)	3 (37.5%)	2 (100%)	0
Leukopenia	5 (62.5%)	2 (25.0%)	0	0
Neutropenia	4 (50.0%)	2 (25.0%)	1 (50.0%)	0
Headache	3 (37.5%)	0	1 (50.0%)	0
Fever	0	0	1 (50.0%)	0

### Pharmacokinetics

**FIGURE 1:**
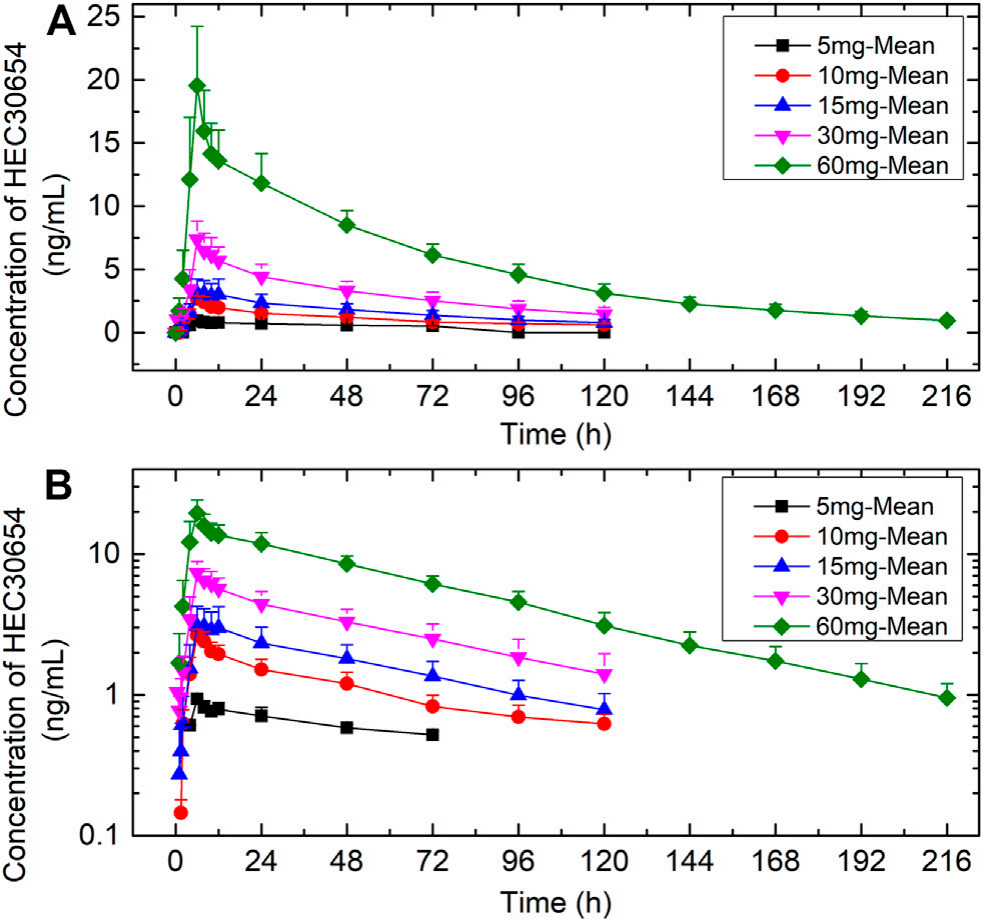
The mean (±SD) plasma concentration-time profiles of HEC30654 after administration of single escalating doses to healthy subjects under fasted conditions. **(A)** linear scale **(B)** semi-log scale.

The mean plasma concentration-time profiles of HEC30654 after administration of single escalating doses to healthy subjects under fasted conditions are shown in Figure 1. Mean PK parameters for HEC30654 are summarized in Table 4. Median t_max_ of HEC30654 was 6 h (range, 4–12 h), t_1/2_ of 10–60 mg HEC30654 ranged from 52.1 to 63.8 h, and there was no dose proportional increase (t_1/2_ of 5 mg HEC30654 was not included in the analyses due to high variability). The CL/F of HEC30654 was 31.8–63.1 L/h with a large Vz/F 3840–6420 L, suggesting a wide distribution in the body. The AUC_0-∞_ and C_max_ of HEC30654 increased in a dose-dependent manner, but the increases were slightly greater than dose proportional in 60 mg. Dose proportionality over the 5–60 mg HEC30654 dose range was evaluated with a power model. The slope (90%CI) for lnAUC_0-∞_ was 1.17 (1.07–1.28) and lnC_max_ was 0.92 (0.76–1.07). The 90% CI were outside the respective acceptance intervals of 0.86–1.14 and 0.91–1.09; however, as the 90%CI of the slope for lnC_max_ contained the value of 1, dose proportionality may be claimed for that parameter.

**TABLE 4:** Pharmacokinetic profiles of HEC30654 and HEC93263.

Compound	PK parameter	5 mg (n = 3)	10 mg (n = 8)	15 mg (n = 8)	30 mg (n = 8)	60 mg (n = 8)
HEC30654	t_1/2_ (h)	140.07 (108.4)	63.77 (33.4)	53.55 (25.5)	55.25 (34.9)	52.1 (12.3)
	*T_max_ (h)	6 (6–6)	6 (6–8)	6 (6–12)	6 (6–8)	6 (4–6)
	C_max_ (ng/ml)	0.937 (1.2)	2.65 (9.9)	3.24 (35.1)	7.31 (18.3)	20 (20.3)
	AUC_0-t_ (h*ng/mL)	39.6 (12.2)	117 (26.2)	185 (29.2)	359 (25)	1100 (15.6)
	AUC_0-∞_(h*ng/ml)	157 (74.2)	177 (29.6)	247 (28.6)	475 (36.1)	1170 (15.8)
	Vz/F(L)	6,420 (23)	5190 (20.1)	4,700 (31.7)	5030 (19.6)	3,840 (16.9)
	CL/F (L/h)	31.8 (74.2)	56.5 (29.6)	60.8 (28.6)	63.1 (36.1)	51.1 (15.8)
HEC93263	t_1/2_ (h)	−	−	−	−	30.3 (60.6)
	*T_max_ (h)	−	−	−	−	6 (4–6)
	C_max_ (ng/ml)	−	−	−	−	1.77 (47.7)
	AUC_0-t_ (h*ng/mL)	−	−	−	−	33 (140.6)
	AUC_0-∞_(h*ng/ml)	−	−	−	−	59 (98.2)

T_max_, time to peak plasma concentration; C_max_, peak plasma concentration; AUC_0-t_, area under the plasma concentration-time curve from time zero to time t; AUC_0-a_, area under the plasma concentration-time curve from time zero to infinity; t_1/2_, terminal elimination half-life; Vz/F, apparent volume of distribution; CL/F, apparent clearance; CV%, percentage coefficient of variation.

HEC93263 exposure was low. Metabolite PK parameters could only be calculated for 60 mg HEC30654 (Table 4). Median t_max_ of HEC93263 was 6 h and t_1/2_ was 30.3 h. The rate of HEC30654 metabolism to HEC93263 was low, as suggested by C_max_ (8.9%) and AUC-_0–∞_ (3%) values.

HEC30654 and HEC93263 were detected in urine and fecal samples. The percentage cumulative recovery of HEC30654 in the urine and feces from 0 to 216 h was 0.998 and 21.4%, respectively. The percentage cumulative recovery of HEC93263 in the urine and feces from 0 to 216 h was low (<0.1%) (Table 5).

**TABLE 5:** Cumulative recovery of HEC30654 and HEC93263 in urine and feces.

		HEC30654 (n = 8)	HEC93263 (n = 8)
Urine	Ae_0-216_ (mg)	0.599 ± 0.221	0.005 ± NC
	%Ae_0-216_ (%)	0.998 ± 0.368	0.008 ± NC
Feces	Ae_0-216_ (mg)	12.811 ± 7.975	0.008 ± 0.008
	%Ae_0-216_ (%)	21.351 ± 13.291	0.014 ± 0.013

## Discussion

This Phase I clinical trial was designed to characterize the safety, tolerability, and PK of HEC30654 in healthy Chinese volunteers.

HEC30654 was safe and well-tolerated in preclinical animal studies. The non observed adverse effect level (NOAEL) in rat and dog were 34.5 mg/kg and 2.3 mg/kg, corresponding to maximum recommended starting doses (MRSD) of 29.2 and 6.3 mg in humans, considering a safety factor of 10. The initial effective dose in rats was 1.5 mg/kg/day, and according to physiologically based PK modeling, the initial effective dose in humans was 5 mg/day. The maximal tolerated dose (MTD) in rat and dog were 690 mg/kg and 1,150 mg/kg, corresponding to MTDs of 6,624 mg and 37,260 mg in humans. Based on these findings, this study planned to randomize healthy subjects to receive a single oral dose of 5 10, 15, 30, 60, 90, or 120 mg HEC30654 or matching placebo. However, the 60 mg HEC30654 treatment group met the pre-defined stopping rules specified in the protocol; therefore, the study was terminated early.

In this study, single doses of HEC30654 up to 30 mg were generally safe and well tolerated, but dose escalation was stopped at 60 mg based on the occurrence of drug-related AEs. All the subjects in 60 mg group occurred grade ≥2 drug related AEs, and three of them occurred grade ≥3 drug related AEs. TEAEs most frequently reported by subjects administered 60 mg HEC30654 included lymphocytopenia, leukopenia, elevated C-reactive protein, neutropenia, fever, headache, and myalgia. The incidence of these TEAEs in the other treatment groups was rare.

The TEAEs reported by the patients administered 60 mg HEC30654 were inconsistent with the AEs observed in patients receiving other 5-HT6 receptor antagonists (e.g., LuAE58054 and SUVN-502: dizziness, aggression, diarrhea, headache, hypertension, vomiting, and nausea (Wilkinson et al., 2014; Nirogi et al., 2018; Frölich et al., 2019; Matsunaga et al., 2019)), findings from preclinical toxicology studies of HEC30654, and the TEAEs reported by the patients administered 5–30 mg HEC30654. Furthermore, these TEAEs were also reported by subjects administered matching placebo.

The TEAEs reported by patients administered 60 mg HEC30654 or matching placebo manifested as concentrated outbreaks, with similar symptoms, and self-limiting processes. Various viral antibodies were detected, but their titer suggested no clinical significance. Other subjects and staff in the same setting showed no symptoms, so the relevance of these findings is unclear.

After single administration, the half-life of HEC30654 ranged from 52.1 to 63.8 h making HEC30654 compatible for once-daily dosing. In comparison, in a Phase II trial, the 5-HT6 receptor antagonist LuAE58054 required thrice daily dosing (Wilkinson et al., 2014; Atri et al., 2018). Exposure to HEC30654 across the dose range included in this study almost increased in proportion to dose. Metabolism of HEC30654 to HEC93263 was slow (<10%), and HEC30654 was mainly eliminated unchanged through feces, which is consistent with *in vitro* findings.

## Conclusion

HEC30654 exhibited an acceptable safety, tolerability and pharmacokinetic profile in healthy subjects in the dose range 5–30 mg. Based on preclinical efficacy in various models of cognition, HEC30654 may represent a therapeutic option for symptomatic treatment of cognitive disorders.

## Data Availability

The original contributions presented in the study are included in the article/supplementary material, further inquiries can be directed to the corresponding author.
